# Translating a health service intervention into a rural setting: lessons learned

**DOI:** 10.1186/s12913-016-1302-0

**Published:** 2016-02-18

**Authors:** Elsa Dent, Elizabeth Hoon, Alison Kitson, Jonathan Karnon, Jonathan Newbury, Gillian Harvey, Tiffany K. Gill, Lauren Gillis, Justin Beilby

**Affiliations:** Discipline of Public Health, School of Public Health, The University of Adelaide, South Australia, Australia; Centre for Research in Geriatric Medicine, The University of Queensland, Queensland, Australia; School of Nursing, The University of Adelaide, South Australia, Australia; Discipline of Rural Health, School of Medicine, The University of Adelaide, South Australia, Australia; Discipline of Medicine, School of Medicine, The University of Adelaide, South Australia, Australia; Vice Chancellor, Torrens University, Victoria Square, Adelaide, South Australia Australia

**Keywords:** Rural health services/standards*, South Australia, Rural health services/utilisation, Knowledge translation

## Abstract

**Background:**

Limited research exists on the process of applying knowledge translation (KT) methodology to a rural-based population health intervention.

**Methods:**

This study reports on the implementation and translational stages of a previously described Co-creating KT (Co-KT) framework in the rural town of Port Lincoln, South Australia (population: 14,000). The Co-KT framework involves five steps: (i) collecting local data; (ii) building stakeholder relationships; (iii) designing an evidence-based intervention incorporating local knowledge; (iv) implementation and evaluation of the intervention; and (v) translating the research into policy and practice. Barriers and enablers to the overall Co-KT implementation process were identified. Our intervention focused on musculoskeletal (MSK) conditions.

**Results:**

Although the Co-KT framework was valuable in engaging with the community, translating the final intervention into daily clinical practice was prevented by a lack of an accessible policy or financial framework to anchor the appropriate intervention, a lack of continued engagement with stakeholders, access problems to general practitioners (GPs) and Allied Health Professionals; and the paucity of referrals from GPs to Allied Health Professionals. Consequently, while many aspects of the intervention were successful, including the improvement of both function and pain in study participants, the full implementation of the Co-KT framework was not possible.

**Discussion:**

This study implemented and evaluated a Co-KT framework for a population with MSK conditions, linking locally generated health care system knowledge with academic input. Further policy, health system changes, and on-the-ground support are needed to overcome the identified implementation challenges in order to create sustainable and effective system change.

## Background

The Australian Health Care System is currently going through an unprecedented structural reform, which in time, will see primary health care services gaining a much more central role [1]. This reform aims to improve the access to health care services by all Australians. Currently, access to health care servies in Australia is inadequate for certain at-risk populations [2], including rural communities. Rural areas are often faced with a shortage of health professionals [3], a lack of communication between health care providers [4], and are located a long distance from tertiary and specialised health services [4]. Subsequently, health care in rural settings tends to be fragmented. Rural dwelling populations also show a higher need for primary health care services than urban populations, predominantly due to their demographic profiles and higher incidence of chronic disease and disability [5].

For effective, sustainable health service improvement, it is reasonable to propose that multi-level health system change is needed. This change can stem from developing programs and policies for primary health care service integration [6]. Recently, a ‘models of care’ (MoC) approach to health system change intervention has been proposed for this exact purpose [6]. The MoC approach involves tackling community need, be it current or projected, and then implementing a framework/evidence-based policy in the community [6]. Whilst the MoC approach is effective at helping health professionals improve health care delivery across Australia, the next challenge is to engage health consumers and other stakeholders in the implementation process. For a more participatory involvement of stakeholders, an implementation framework based on Knowledge Translation (KT) is well suited [7]. KT has been referred to as action-oriented, collaborative research that is designed to fill the gap between what is known regarding evidence-based practice and what is actually done [7, 8].

KT is a concept gaining much international momentum in health systems research [9, 10]. In Australia, the importance of using KT frameworks for population health interventions is being increasingly recognised. Our research group previously developed a ‘Co-creating of KT’ (Co-KT) framework, which combines academic evidence-based knowledge with the context-specific knowledge from stakeholders [11, 12]. The Co-KT framework is a useful guide for our rural population health intervention, given that it promotes collaboration amongst multidisciplinary health professionals whilst still ensuring that elements essential for knowledge translation (such as strategic management of stakeholder relationships) are present at all stages of the intervention [11]. Recent research has highlighted that the success of a health system intervention hinges on the full engagement of relevant stakeholders, and the appropriate use of their knowledge of their community and local health care system [1]. That is, stakeholder collaboration at all stages of a population health intervention will maximise the chances of reducing inequalities in regards to access to health care services [13].

In our Co-KT framework, relevant stakeholders were involved at all stages of the population health intervention. Specifically, the Co-KT framework has five steps: (i) collating local knowledge, including identifying opportunities for better health care integration and improvement; (ii) building stakeholder collaboration; (iii) using this locally generated knowledge to design an evidence based intervention; (iv) implementing and evaluating the intervention; and (v) achieving and sustaining effective system change [11, 12].

The aim of this paper was to: (i) describe the practicalities and lessons learned from implementation of a population health intervention study in a rural setting using a Co-KT framework as a guideline for intervention; and (ii) provide an insight into the barriers and enablers encountered during the study’s implementation.

### Musculoskeletal conditions (MSK)

Musculoskeletal conditions (MSK) were selected as the group of medical conditions for implementation of the Co-KT framework. MSK conditions encompass low back pain, osteoarthritis, rheumatoid arthritis, osteoporosis, muscle strains and any other damage to the musculoskeletal system [5, 6]. There were multiple reasons why MSK conditions were selected as the focus of our population health study:

- It was the most common condition identified in our LINKIN health census, affecting 42 % of the Port Lincoln population aged ≥ 15 years [5].
- MSK conditions are a national health priority in Australia, however the development and implementation of solutions to alleviate MSK related morbidity remains largely unrecognised in existing Australian health care policies [6].
- In Australia, the leading cause of disability is acknowledged to be MSK conditions, affecting around one third of the Australian population [6]. The prevalence of MSK conditions found in our Port Lincoln health census was much high than that in the general Australian population [5].
- Consultations with local service providers identified issues around coordination and access to services for people with MSK conditions.
- Local employment in Port Lincoln is dominated by agricultural and fishing industries, which require good physical mobility of employees.
- MSK conditions place a heavy burden on the health care system and individuals, both financially and on their quality of life [14–17]. MSK conditions are also are linked to a range of co-morbidities and a poor state of self-rated health [18].
- Access and use of physiotherapists tends to be low in rural locations [19].
- There was limited understanding of how different subpopulations with MSK conditions interact with the health system to manage their condition [5, 18].

## Methods

This study reports results from the implementation and translational stages of the LINKIN population health study. The LINKIN health study evaluated the functioning of the health system in the rural fishing and farming community of Port Lincoln, South Australia (population: 14,000). Port Lincoln is classified as ‘Outer Regional Australia’ by the State Accessibility/Remoteness Index of Australia (SARIA+) (SARIA+ score of 5.79) [20].

### The contextual framework for implementation of the LINKIN health study: co-creating knowledge translation (Co-KT) framework

The Co-KT framework was used as an implementation guide at all stages of our LINKIN health study. The five steps of implementation are discussed in this section.

### Step 1: collating local knowledge, including identifying opportunities for better health care integration and improvement

The first step of the Co-KT framework was to identify opportunities for locally relevant service improvements to address unmet needs and equity issues for the Port Lincoln community. This phase generated a quantitative snapshot of both health and health service use in the local community by way of a population health census, a Computer Assisted Telephone Interview (CATI) and qualitative community dialogue on the purpose and value of the LINKIN study.

The population health census was conducted in 2010, and collected local data regarding health status, socio-economic position, health-related quality of life (QOL) and access to health services. We have described the methodology of our health census previously [21]. Importantly, the census contained a component to identify respondents who were willing to be re-contacted about further involvement in the study. Informed, written consent to participate in the LINKIN study was required for all participants.

From the health census, a number of conditions were identified as having a high burden in Port Lincoln, including cardiovascular diseases, diabetes, mental distress, and musculoskeletal (MSK) conditions. We focused primarily on MSK conditions for our LINKIN health study.

Respondents who self-reported a MSK condition in the health census and who agreed to recontact were invited to complete a CATI survey in 2012, beginning with the question: “do you currently have any bone or joint problems?” [5]. The CATI survey focused on gaining a process-based understanding of the complexities of patient movement across the health care system, and included questions on the following topics: self-management activities, health care preferences, reasons for primary health care use, the sequence of health providers visited (if any), QOL, details of the specific MSK condition/s, and reasons for change in the use of service providers (if any) [5].

### Step 2: building stakeholder collaboration

Two main strategies were used to build stakeholder collaboration:

- Two-way information sharing through meetings with local communities, businesses, information groups and service providers. For instance, relevant local stakeholders contributed valuable contextual information, and researchers brought knowledge on evidence-based practice in population health interventions.
- Media dissemination to increase awareness of the LINKIN project in the local community—radio interviews, radio and television advertisements, newspaper articles, regular LINKIN newsletters outlining progress of the study.

### Step 3: using locally generated knowledge to design an evidence-based intervention

Step 3 of the Co-KT framework identified potential intervention strategies using knowledge gained during the first two steps of the Co-KT framework. Firstly, a literature review was performed to identify applicable interventions, with data extracted to inform the following decision-making criteria to evaluate alternative intervention options:

- Cost-effectiveness
- Feasibility and likelihood of benefit—proportion of eligible population likely to benefit (High/moderate/low)
- Magnitude of effect for persons benefitting from the program (High/moderate/low)
- Long-term sustainability of program (High/moderate/low)
- Population size: maximum number of events to be prevented (prevention)/number of individuals using program (self-management/clinical practice)
- Feasibility of recruiting patients (High/moderate/low)

### Step 4: Co-KT evaluation

Identified barriers and enablers to implementing the Co-KT framework were classified according to de Goede’s KT methodology [22], as applied by Goyet et al. [23]. Two types of barriers/enablers were identified: *process* level factors, and *individual* factors [22]. Process level factors are components that can be altered during the implementation process of the KT framework [22], whereas individual factors are more difficult to change, and are related to stakeholder values and the study context. Barriers and enablers to Co-KT framework implementation with regards to the collaboration *between* stakeholders and researcher were also identified.

### Step 5: achieving and sustaining effective health system change

The final step of the Co-KT framework involved an assessment of the impact of the intervention on the community, and the sustainability of the intervention beyond the evaluation phase.

### Ethics statement

Ethics clearance was granted for this project by the University of Adelaide Human Research Ethics Committee (Protocol Number: H-036-2010). The project adhered to ethical guidelines from the Australian Code for the Responsible Conduct of Research. All participants in the study signed informed consent.

## Results

**Fig. 1:**
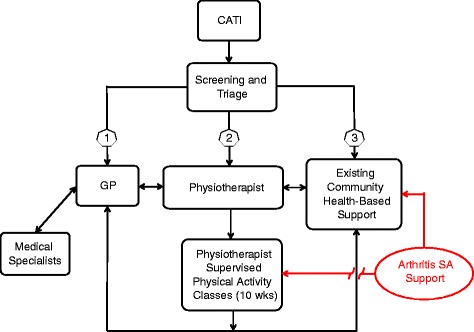
Flow diagram of the Self-Management Plus intervention for the LINKIN health study (CATI = Computer Assisted Telephone Interview)

Through meetings and ongoing liaison with stakeholders, we co-designed a population health intervention to address the unmet health service needs of people with MSK conditions residing in Port Lincoln (see Fig. 1). This intervention included a triage service, which included referral to physiotherapists, GPs, and community health-based self-management and physical activity options. Barriers and enablers affecting project implementation at both the *process* and *individual* level are detailed in Tables 1 and 2 respectively. Table 3 outlines the barriers and enablers to stakeholder-researcher collaboration.

**Table 1:** Process Level Factors Influencing the Implementation of the Co-Creating Knowledge Translation (Co-KT) Framework in the LINKIN study

Process Factors	Enablers	Barrier
Preparation Phase of the Research		
Insight into the working process	• Use of the Co-KT framework by researchers allowed the collation of locally generated knowledge, including identifying opportunities for better health integration and improvement.	• Researchers were not fully aware of the on-the-ground policy process of stakeholders in Port Lincoln—the CATI and health census data did not identify on-the-ground issues to the working processes. • The working process in Port Lincoln changed early in the intervention, with key stakeholders changing. • The local infrastructure was lacking with regards to health services: o There was a shortage of physiotherapists. o There were no accredited Exercise Physiologists, resulting in a lack of expertise in clinical exercise interventions for people with chronic and/or complex medical conditions or injuries. o There were limited networks between local Allied Health and GP services, which in turn, inhibited patient referrals.
Study Design	• The longitudinal nature of the study meant that it was possible to compare participant outcomes in the intervention and wait-list groups. • Referral forms were implemented to aid patient referral: o For GPs to refer to physical activity and/or physiotherapist treatment. o For physiotherapists to refer to Physical Activity centres.	• Due to the longitudinal nature of the study design, and over 6 months between patient identification and re-contact, many participants no longer had musculoskeletal problems when recontacted by researchers to take part in the intervention phase of the project. • With the high number of co-morbidities of LINKIN participants, the majority of participants required a GP clearance prior to their physical activity/physiotherapy participation. Given that the waiting list for GPs was up to 8 weeks during our study period, there was a considerable delay in implementing our study intervention.
Policy Process	• There are some policies for people with musculoskeletal conditions in Port Lincoln, but these were for people with Health Care Cards (low income earners) only.	• Mid-intervention saw both the state and federal government changes in health policy, affecting health service usage in Port Lincoln—including closing Medicare Locals and re-shuffling Country Health South Australia infrastructure. • Musculoskeletal problems were identified as the main problem in Port Lincoln, but this was not a priority are in the State Government’s 10 Year Local Health Service Plan for Port Lincoln.
Degree of Uncertainty	• We employed a physiotherapist to run our intervention “on-the-ground” who was able to provide us with proactive information, which reduced the level of uncertainty in the project.	• The fitness centre climate in Port Lincoln is continually evolving: during the year-long intervention, 2 fitness centres had changed owners, and a further 2 were established.
Timetable	• The project was flexible, so we were able to implement the study within the 12-month timeframe.	• The local football season coincided with the middle of our intervention study, resulting in long waiting lists of study participants to see physiotherapists. • The changes in study design, due to an abrupt change of location and stakeholder needed, meant a 2-month delay in recruiting participants. • There were many attendance disruptions as many participants were retirees and often travelling frequently for long periods.
Transfer stage of the Research		
Media	• Researchers publicised the LINKIN study, through regular newsletters to the community /stakeholders as well as newspaper articles. A “LINKIN” logo was present on all media to improve the credibility and recognitition of the research project.	• The website for the project was not permanent.

**Table 2:** Individual Level Factors Influencing the Implementation of the Co-Creating Knowledge Translation (Co-KT) Framework in the LINKIN study

Individual Factors	Enablers	Barrier
Perceived Robustness and credibility of the research.	• Researchers used local knowledge gathered from: (i) a population health census; (ii) a CATI; and (iii) stakeholder meetings • The research was published in peer-reviewed journals [5, 11, 12].	• Researchers were unable to ascertain how the robustness of the LINKIN Health Census and CATI results were received by stakeholders.
Fit with Belief Systems	• Musculoskeletal conditions were chosen for the intervention for PL, given they were the highest co-morbidity in the population.	• Local GPs were seemingly unaware of the specialist skills of Allied Health Professionals. They were also reluctant to refer to Allied Health Professionals as they were not convinced of their benefit. • Local small town alliances were hard difficult to work with—eg referrals from GPs directly to chiropractor clinic, in preference to a physiotherapist. • Patients often had multimorbidities, including cardiovascular disease. These multimorbidities took priority over musculoskeletal conditions.
Prioritising Problems	• Meetings with stakeholders provided researchers with local information on which problems to prioritise.	• Musculoskeletal conditions were not seen as a priority by stakeholders.
Responsibility	• Researchers followed the Co-KT framework, which involved setting up key roles for stakeholders.	• Key roles for stakeholders were unable to be established. The responsibility for implementation of the research fell predominantly on the researchers.
Consideration of which issues were at hand	• A member of the research team was a GP in Port Lincoln, which gave us updated information of current issues.	• The continually changing environment for Allied Health Professionals at Port Lincoln meant that it was not easy for researchers to readily identify newly forming barriers. • Costs for chiropractors and alternative health practitioners were less than that of physiotherapists. • There was a low engagement of patients with regards to care management of their musculoskeletal condition.

**Table 3:** Barriers and Enablers to Stakeholder-Researcher Collaboration during the LINKIN study

Enablers	Barriers
• A local GP was a member of the research team, serving to bridge the gap between researchers and the community. • There was a high level of interest by stakeholders at the beginning of the project. • Researchers invited local GPs and Allied Health Professionals along to workshops to discuss the design of the intervention.	• Researchers found it difficult to influence the policies and structures within the bureaucratic-structure of local stakeholders. • Ongoing iinterest in meetings was low for stakeholders, and we only had a limited number of GPs and Allied Health Professionals in attendance. • Interactions between researchers and stakeholders was limited due to both time and distance constraints.

### Lessons learned

Our study uncovered several important learning points. These learnings included: (i) strategies for overcoming intervention barriers; (ii) the importance of a flexible intervention framework; and (iii) facilitating sustainability through the continual engagement of stakeholders. These learnings are discussed below. In addition, Table 4 outlines lessons learned from the study and their practical implications.

**Table 4:** Recommendations and Implications for Implementation of a Population Health Study: Learnings from LINKIN

Recommendations
• Policy makers and funders in the local community should be kept very heavily and actively engaged throughout the project.
• Both researchers and stakeholders need to be aware of each other’s priorities in implementing the Co-KT framework.
• Timelines need to be flexible and account for local community issues, such as access problems to local health services.
• Researchers need to have all organisational levels of a stakeholder engaged, not just the on-the-ground staff and the high management staff. Prior assessment of the Organisational Readiness to Change would facilitate this knowledge.
• Inter-professional education will help to promote networking amongst healthcare providers.
• The intervention should focus not only on those participants who are engaged with the health care system, but also on participants who are not yet engaged.
• Medical support should be coupled with psychosocial support for people with musculoskeletal problems, and these support services should be integrated.

#### (i) Strategies for overcoming intervention barriers

Strategising to overcome intervention barriers was a prevailing learning point in our study. When rolling out our Co-KT framework, we found that there was a long waiting list for both GPs and Allied Health Professionals, which in turn, was compounded by the majority of patients needing to consult a GP prior to their participation in a physical activity program. To overcome this challenge, our research team employed a local physiotherapist to run our intervention, which vastly sped up the study intervention. Moreover, this physiotherapist was able to provide us with practical on-the-ground feedback in order to overcome day-to-day challenges the project faced. Also of note, we found that there was a lack of GP referrals to Allied Health Professionals in Port Lincoln. We strategized with local stakeholders to develop a specific referral form for GPs to use, which assisted in the referral of study patients to our physical activity intervention [24].

#### (ii) Flexible intervention

The importance of a flexible intervention was also an important learning point in our study. We found that we needed to use the Co-KT framework in a dynamic and flexible manner during our study. For example, our initial intervention location was under renovation immediately prior to patient recruitment, and we were required to use stakeholder feedback to find a new suitable study location. We found that closer engagement of our key stakeholders, through regular one-on-one meetings, allowed us to shape a more flexible intervention. We found that proactively identifying barriers to our intervention roll-out allowed us to pin-point issues as early as possible.

#### (iii) Facilitating sustainability through the continual engagement of stakeholders

An additional key learning point in our study was the inherent difficulty in maintaining engagement of stakeholders. We were unable to convince the local stakeholders of insights provided by the CATI and Census data analyses on gaps in available services and the unmet needs of patients with long term MSK, and a proposed model of care in which all stakeholders use a single point of entry (physiotherapist triage) was not acceptable to all stakeholders.

Moreover, we found that although many stakeholders were involved in the planning stages of the project, they later did not engage during the intervention phase of the project. This lack of long-term stakeholder engagement existed despite running regular project meetings and media awareness campaigns (such as project newsletters). We found that whilst researchers were able to facilitate buy-in from the management level of local organisations, it proved difficult to influence the policies and structures within the organisation themselves. This issue was commonly related to changes in personnel and leadership priorities within government agencies. It has been a time of change within the Australian Health Care System, with new interventions being installed and a restructure of Country Health South Australia and Medicare Locals, and new local private providers coming on board. As such, although we were able to involve and integrate local providers for the duration of the study, we were not able to develop a sustainable long term shared plan to enable leveraging of existing assets to ensure the sustainability of the evaluated intervention beyond the funding of the research project.

## Discussion

This longitudinal population health research project used a Co-KT population health framework to design and implement an intervention program for musculoskeletal conditions in a rural area. Whilst treatment and management guidelines for MSK conditions are well established [6], little research has been conducted on procedures for implementing these guidelines at the population level in a rural community. This study is also one of the first to look at operational characteristics of an intervention for MSK conditions. A further unique aspect of the present study is that participants for the study were identified from a large-scale population health census with over 8,000 respondents.

This study found that whilst the Co-KT framework itself allowed for linking of locally generated health care system knowledge with academic input, it was not possible to reach the sustainability/embedment stage of the framework. Multiple barriers encountered during our intervention hindered this progression towards a sustainable intervention. These barriers were of two main types: those related to the roll-out *process* of our Co-KT study, and those related to the *individual* factors related to our stakeholders and study context.

Unsustainability is common issue encountered by many KT implementation studies worldwide [25, 26]. It is likely that the success of a Co-KT intervention is contingent on the implementation climate at the organisation level [27], as well as the motivational readiness and resources of the stakeholder organisation itself [28]. Indeed, a low level of organisational readiness to change has been identified to be a major barrier to effective KT intervention [28]. Of particular note, a difficulty in continued stakeholder collaboration has been higlighed as a major barrer to specifically managing populations with MSK conditions [29]. Finally the changing models of primary care in Australia (which includes the transition from Medicare Locals (primary health care organisations) to Primary Health Networks) and Country Health partnerships prevented the establishment of a possible future sustainable program in our study.

Collaborative relationships with key stakeholders in the community are vital to sustainability of a Co-KT framework [12]. This collaboration needs to ensure both engagement and integration of local providers. We purposely planned our Co-KT framework for the LINKIN health study to involve stakeholders at each step of the framework, rather than simply implementing our research findings directly into the community [12]. However, even though we had the advantage that a member of our research team was a local GP, we were not able to continually engage stakeholders.

Our results, although disappointing, are not unusual. For example, despite over a decade of research into KT implementation in population health, there still remains large evidence-practice and policy gaps [7], particularly in rural Australia [5]. As a research team we worked to engage all local stakeholders, adapt to local service priorities and analyse what evidence based interventions were suitable for a rural community. Eventually the lack of access to health service providers and an adaptable local policy framework prevented the long term sustainability of the project. What we lacked were on-the-ground project facilitators in Port Lincoln who could work systematically, locally and constructively on the barriers identified to implementation. Future studies should focus much more strongly on developing constructive relationships at the service delivery level, and at different levels of policy, coupled with financial incentives and infrastructure changes.

## Conclusions

This study implemented a Co-KT framework to develop and improve service provision for areas of unmet need in a rural Australian population, linking locally generated health care system knowledge with academic health research. The Co-KT framework was a valuable working model, but it was necessary to use the framework in a dynamic and flexible way due to the implementation barriers encountered during the intervention. Several barriers to implementation and sustainability of the project were identified, including access problems to GPs and Allied Health Professionals in the local community and the lack of a long term sustainable policy and financial framework.

## Abbreviations

CATI: Computer Assisted Telephone Interview
Co-KT: co-creating knowledge translation
KT: knowledge translation
LINKIN: shortened ‘nickname’ for our population health study in Port Lincoln
PL: port Lincoln
QOL: quality of life
